# Management of children with autism spectrum disorder in the dental setting: Concerns, behavioural approaches and recommendations

**DOI:** 10.4317/medoral.19084

**Published:** 2013-08-29

**Authors:** Konstantina Delli, Peter A. Reichart, Michael M. Bornstein, Christos Livas

**Affiliations:** 1 Oral Medicine and Pathology Specialist and Researcher, Department of Oral and Maxillofacial Surgery, University of Groningen, University Medical Centre Groningen, The Netherlands; 2 Visiting Professor, Department of Oral Surgery and Stomatology, School of Dental Medicine, University of Bern, Switzerland; 3 Senior Lecturer, Department of Oral Surgery and Stomatology, School of Dental Medicine, University of Bern, Switzerland; 4 Consultant, Department of Orthodontics, University of Groningen, University Medical Centre Groningen, The Netherlands

## Abstract

Objectives: This article reviews the present literature on the issues encountered while coping with children with autistic spectrum disorder from the dental perspective. The autistic patient profile and external factors affecting the oral health status of this patient population are discussed upon the existing body of evidence. Material and Methods: The MEDLINE database was searched using the terms ‘Autistic Disorder’, ‘Behaviour Control/methods’, ‘Child’, ‘Dental care for disabled’, ‘Education’, ‘Oral Health’, and ‘Pediatric Dentistry’ to locate related articles published up to January 2013. Results: Most of the relevant studies indicate poor oral hygiene whereas they are inconclusive regarding the caries incidence in autistic individuals. Undergraduate dental education appears to determine the competence of dental professionals to treat developmentally disabled children and account partly for compromised access to dental care. Dental management of an autistic child requires in-depth understanding of the background of the autism and available behavioural guidance theories. The dental professional should be flexible to modify the treatment approach according to the individual patient needs.

** Key words:**Autism spectrum disorder, dental management, children.

## Introduction

Autism spectrum disorder (ASD) refers to a group of neurodevelopmental disabilities with a core set of defining criteria that comprise impaired social interaction, communication, and restricted or repetitive behavioural stereotypes. The spectrum consists of autism, Asperger Disorder (AD), and Pervasive Developmental Disorder-Not Otherwise Specified (PDD-NOS), which differ in the number and severity of diagnostic features (1).

The etiologic background of ASD, though not yet completely understood, is considered to implicate both genetic and environmental factors. Recent research work has elucidated that parameters such as CNTNAP2 gene, de novo mutations, mitochondrial defects (2), cytosine dysregulation, high maternally derived intrauterine androgen concentrations, and advancing maternal age (3) may be involved in the pathophysiology of autism. Based on lately published data (4), ASD prevalence underwent a substantial increase within a decade of surveillance, estimated at 11.3 per 1,000, i.e. one in 88, children aged 8 years. In addition, a male: female ratio of 4.6: 1 has been reported on the gender-specific epidemiology of the autistic disorder.

The symptomatology of ASD initiates before the third year of age and generally undergoes a steady course without remission through aging (5,6). Established features in the autistic child such as marked impairment in the use of multiple nonverbal communications, failure to develop social relationships and share experiences and interests, delay or complete lack of linguistic development as well as inflexible adherence to rituals (7), potentially coexisting with sensory disabilities, mental retardation or epilepsy (6), may hinder professionally delivered and home dental care placing individuals with ASD at high risk for oral diseases.

Furthermore, the psychological well-being of parents of a child with diagnosed ASD is significantly influenced by the behavioural difficulties of their offspring. Synopsis of the existing literature reveals accentuated stress, more psychological distress and depressive signs, lowered living standards and increased rates of physical and mental health problems in guardians of autistic children (7). Upbringing a child with ASD generates stressful conditions which in most cases are associated with adaptation to child’s routine, interference with education and health care systems, coordination of multidisciplinary caregivers, and limited availability of resources (8). Subsequently, a scheduled dental visit may represent a major ordeal for all parties involved; children with autism, parents and care providers.

A MEDLINE (www.ncbi.nlm.nih.gov/pubmed) search was conducted using alternatively the Medical Subject Headings terms ‘Autistic Disorder’, ‘Behaviour Control/methods’, ‘Child’, ‘Dental care for disabled’, ‘Education’, ‘Oral Health’, and ‘Pediatric Dentistry’ combined with the Boolean operators ‘AND’ and ‘OR’. Our aim was to identify studies on the dental related problematique of children with ASD published up to January 2013. In this review article, aspects requiring attention in the management of autistic children from the dental perspective are summarized.

## Oral health and autism

Conflicting results have emerged by the limited number of studies that carried out normative oral health assessment in children with ASD (1,5,9-16) ( Table 1). In this context, higher caries prevalence has been previously reported for autistic populations compared to other oral conditions (10), non-autistic controls (5), and schizophrenic patients (16). In contrast, lower caries indices in relation to healthy siblings (1,11) and developmentally disabled children (15) have been also assigned to ASD patients. Interestingly, there have been merely two controlled studies with unaffected counterparts that announced statistically significant caries susceptibility for autistic samples, either higher (5) or lower (1). With respect to oral hygiene, the preponderance of publications (5,9-12,14,16) points to rather poor standards in young autistic patients, reaching as well statistical significance (9-12,14). It is noteworthy however, that children and young adults with autism maintained best oral hygiene among special school attendants with other developmental disabilities (13). Moreover, clinical manifestations of orthodontic interest, i.e. anterior open bite and dental crowding, were diagnosed more frequently in adults with ASD than unaffected pairs (12). Likewise, spacing, reverse overjet, open bites and Class II molar relationship tendencies were higher in autistic patients evaluated by orthodontists (14). Presumably, the compromised dental status in conjunction with harmful habits including bruxism, tongue, thrusting, and lip biting often displayed by children with autism (5) may result in certain malocclusions. However, methodological issues such as small sample size, lack of non-autistic control group and variety of assessment tools used alert for cautious interpretation of the results of the aforementioned studies.

**Table 1:**
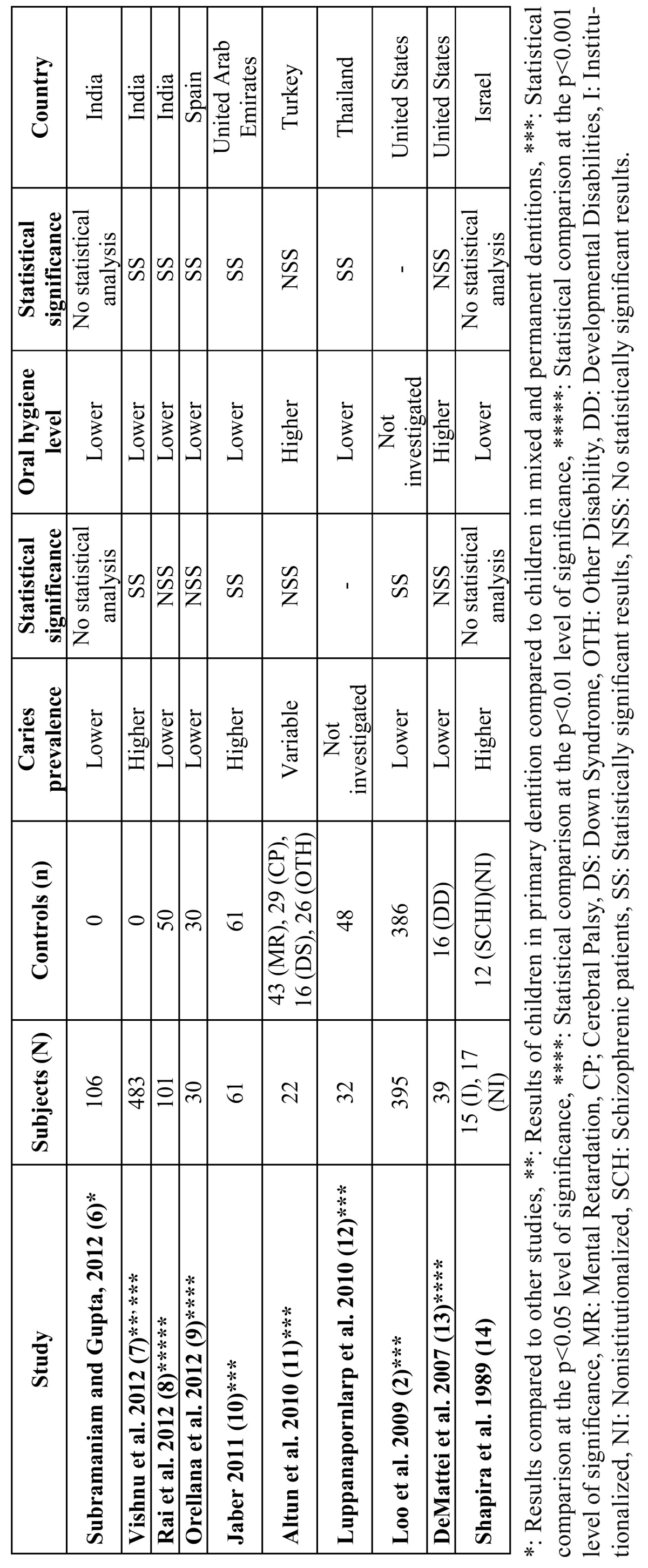
Studies that evaluated the oral health status in autistic patient groups.

The underlying rationale of dental decay prevalence in autistic children has been sought in physicochemical properties of saliva, dietary and oral hygiene habits. Hence, Bassoukou and colleagues (17) concluded that individuals with autism have neither higher salivary flow rate nor superior buffer capacity related to non-autistic controls. Determination of the total antioxidant concentration of salivary samples collected from autistic children revealed significant reduced values compared to normally developed subjects of the same age, which apparently did not affect the caries experience (11). Regular dietary habits with relatively lower frequency of in-between meals snacking and decreased carbohydrate intake have been also claimed to account for the low cariogenic activity in youngsters with autism (18). On the contrary, unfavourable dietary behaviour with persistent preference for sweetened and soft food, and prolonged food retention in the oral cavity has been also described for young autistic patients (5,12). High- otherwise paradoxical - oral health standards observed elsewhere might be also attributed to the dental hygiene routine of children with ASD supervised or performed, on a regular basis, by parents and caregivers.

## Barriers to dental care access

Child’s attitude towards dental procedures, expenditure and lack of insurance coverage have been acknowledged as the main burdens to oral care delivery for children with ASD by a recent large-scale parent survey (19). Aversion to dental treatment, complications associated with the medical condition, and difficulties in locating a practitioner willing to provide care have been further reported by guardians of children with autism and other developmental disabilities (DD) (20). Limited availability of dental specialists trained to serve special need patient groups may also complicate the access of this population to oral health services.

## The impact of undergraduate education

**Figure 1:**
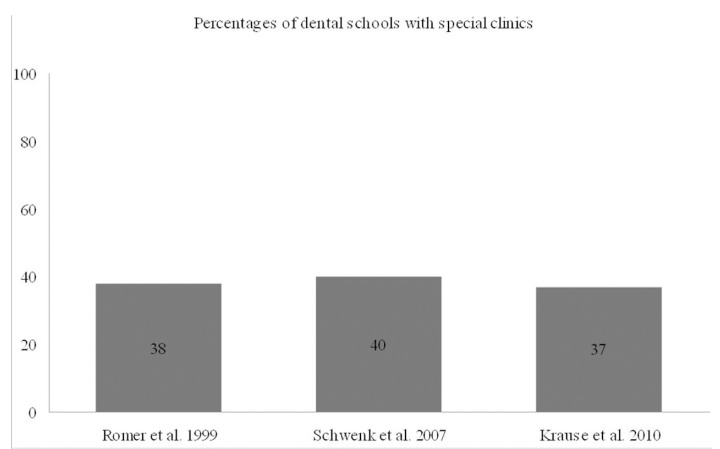
Percentages of dental schools with special clinics reported by assessment studies of dental education.

Apparently, the quality of dental education is highly influential on the professional functioning and self-esteem of the prospective practitioners in treating special needs patients. It is characteristic that more than 60 percent of the general dentists felt not well or not at all prepared to treat patients with special needs and mental retardation (21). In the mid 2000’s the Commission on Dental Accreditation in the United States released a statement, which enforced the competence of dental students in assessing the treatment requirements of patients with special needs. Since then, certain steps were taken to improve the undergraduate dental education concerning the treatment of underserved patient groups. Thus, the total of U.S. and Canadian dental schools responding in a questionnaire study incorporated theoretical and clinical courses on special needs patient care. Equally important, dental residency training has been demonstrated to address the needs of ASD patients in 20 out of 22 institutions (22). Another positive finding was that DD patients have been receiving treatment at all dental faculties with special clinic areas interviewed by Schwenk and colleagues (23). Viewed from a different perspective, the number of schools with designated special patient care clinics remained practically unchanged through the last years (22-24) (Fig. 1). Across special clinical accommodations, prerequisite classes are still not considered by the vast majority of dental school curricula (22). Newly introduced distance learning modes may compensate for undergraduate educational discrepancies, as it has been indicated by late research work; cognitive perception of health professionals about autism interventions has been increased using Internet-delivered programs (25).

To conclude, relevant research has been so far conducted using properly structured but heterogeneous questionnaires that do not allow for direct comparisons between studies. Theoretically, increase of didactic and clinical hours in the predoctoral dental education program can be translated into more effective management of the DD population at the private practice level of care, and this will eventually eliminate disparities in the overall provision of dental care. In the future, it will be interesting to carry out questionnaire-based surveys addressed to faculty members, undergraduate students and dental professionals deriving from the same institutions.

## Autistic behavioural characteristics related to the dental visit

Knowledge and in-depth understanding of basic behavioural patterns is salient in successfully coping with a child with ASD at the dental office. One of the earliest indicators of the autistic disorder is the failure to develop joint attention, which literally means lack of curiosity for the environment and incapability of the child to share information using spoken language, gestures and eye contact (6). It is conceivable that lack of responses to demonstrations and inability to establish personal contacts with the personnel may obscure professional oral care proceedings. Impaired sensory perception has been also well-described in the literature for patients with ASD. Thus, malfunction in interpretation of stimulus intake, may result in aberrant responses to visual, auditory, tactile, olfactory and gustatory signals (26). Despite available data suggests a link between socialization and sensory mechanisms in autistic individuals, the chronological order of events has not been explicitly specified yet. In other words, whether the inability of the patient to engage in the environment leads to sensory processing disorders or vice versa remains to be determined. Most importantly, the dental professional during examination should bear in mind that autistic individuals exhibit wide variation in abilities, intelligence, and performance (11). Due to the multifaceted symptomatology of the autistic disorder, practitioners may need to target their therapeutic approach to the unique characteristics of each presenting child.

## Behavioural management approaches

Several basic behaviour guidance methods have been recommended to accommodate dental therapy of autistic patients, including the presence of parents, the use of tell-show-do technique, short, clear commands, and differential verbal reinforcement (1). Application of the visual pedagogy concept or combined use of shaping, reinforcement and sensory adaptation can also enable patients with ASD to undergo dental examination. For a child with restricted receptive skills and lack of joint attention, the use of reward statements may not bring about the desired results during dental treatment (27). Younger autistic children may respond better to certain management techniques such as positive reinforcement. Therefore, the influence of child’s age on social skills might be critical in handling the autistic patient behaviour (19).

A. Visual pedagogy

Identification of the variables that arouse aversive behaviour may contribute in establishing favourable conditions for the autistic child to cooperate at the dental practice. The process known as functional behavioural assessment (28) may take place during the previsit consultation of parents. At that time, the dentist can organize the home-centered preparation that includes familiarization with dental instruments, teaching of skills required for the dental examination using phrases such as ‘open your mouth’, and developing custom-made photo books to assist the child to get acquainted with the dental operatory room (27). The latter model takes advantage of the ability of children to make better contact by means of pictures instead of words (29). In the past, the same technique, defined as visual pedagogy, has been utilized through series of coloured photographs describing step-by-step dental visit and toothbrushing to introduce dentistry and oral hygiene to autistic children (29,30). With regard to oral hygiene, dislike of the taste of toothpaste as well as the feeling of the toothbrush may compromise the effectiveness of either the parent or the child in removing the dental plaque (26). A gentle introduction to toothbrushing using alternatives such as a washcloth, toothbrushes of different texture and design or an electric toothbrush may enhance the acceptance of toothbrush by the child with ASD. Likewise, testing of various toothpastes supervised by the parent or dental professional can be helpful in selecting the one with the most tolerable taste. As a final point, child’s self-protectiveness may be eliminated by intensive behaviour programming, instructed by parents cognizant of reinforcement-based teaching (31).

B. Sensory adapted environment 

The importance of environmental factors in determining the comfort level of children with DD during stressful medical events has been emphasized by Shapiro and colleagues (32). Distraction, aversive reaction and behavioural difficulties may be invoked by loud, unexpected, nearby noises. Presumably, noise disturbances may be exaggerated in busy dental facilities with multiple operating units in the same room. Autistic children may persist to cover their eyes or squint under light exposure, while typically developing counterparts adapt without problems (33). Besides, children with ASD may present hypersensitivity in intraoral and perioral regions, and therefore experience frustration by a light touch or even fall back during dental examination. Thus, physical and verbal aggression, withdrawal or attempt to fight back might be expected from a young autistic patient in the milieu of dental practice as a consequence of aggravated sensory processing (26). Oral defensiveness has been previously evidenced in approximately 50 percent of children with ASD (15).

The dental clinic per se represents an anxiety-provoking environment with bright fluorescent lights, devices generating sharp noises like dental drill, and materials of unfamiliar texture, taste, and smell. Noise disturbances may be exaggerated in busy dental facilities with multiple operating units in the same room (26). Emotional discomfort elicited by surrounding distracting stimuli may be minimized by sensory adaptation of the clinical environment (32). The experimental introduction of relaxing light conditions, rhythmic music, and deep pressure in the dental setting diminished adverse patient reactions and enhanced positive participation in dental prophylactic cleaning (32). It can be asked from parents to bring the child’s favourite music video or music CD. Duration of the dental visit, and sensory sensitization should be kept to a minimum (1). Towards this end, a single operating room may be also reserved to accommodate the treatment of the autistic child. Finally, even while the procedure is in progress, dental specialists and assistants should be consistently concentrated on identifying parameters-triggering points of deviant reactions.

C. Applied Behaviour Analysis (ABA) 

Applied Behaviour Analysis is the branch of psychology that through the analysis of the relationship of behaviour and the environment intends to modify behaviours to achieve desired effects (27). Practice and research across the disciplines of child development, psychology, nursing, and pain management advanced the development of techniques aimed to facilitate children with autism to sustain physical exam, phlebotomy, and intravenous insertion (34). ABA principles have been also adopted in a young autistic patient with needle phobia and diabetes to permit medical monitoring of his blood glucose levels (35). In shaping, through successive approximations of the behaviour intended to be modified, the child is reinforced to adopt the behaviour eventually on his own initiative. With this technique the child can be taught to sit on the dental chair by himself (27). Reinforcement represents one of the elemental behavioural concepts. It is considered to occur when there is an increase in certain behaviour, as a consequence of a stimulus or event following that behaviour. The ‘positive’ or ‘negative’ reinforcement classification depicts whether the increase of the behaviour is linked with initiation or termination of the stimulus respectively (27). For example, giving a sticker, a ‘good job’ or ‘well done’ praise might serve as a positive reinforcer, in case there is proof it leads to enhanced compliance in the dental chair. On the other hand, the child may be negatively reinforced or motivated to stand still during drilling for a predetermined time period, for instance counting from 0 up to 10, if immediately after the intervention is interrupted for a while. The sequence of events is repeated as long as necessary for the procedure to be completed. Using shaping and reinforcement as per case requirements may be beneficial in founding communication with a child with ASD (27).

D. Advanced behaviour guidance methods

Under certain circumstances, dental management of these patients may be performed in combination with advanced behaviour guidance techniques. Antipsychotic medications are most commonly prescribed for ASD patients to alleviate symptoms of irritability, distress, self-injurious behaviour, aggression and delusions (36). The dental specialists should be aware of the oral adverse reactions of the aforementioned drugs, which can be summarized as xerostomia, sialorrhea, sialadenitis, stomatitis, gingival enlargement, edema and discoloration of the tongue (37). The combination of autism behavioural deficits and the nature of the therapeutic intervention necessitate the administration of general anesthesia in approximately 40 percent of cases (36). Advanced behaviour guidance methods such as protective stabilization by means of restricting device and dental staff or parents, conscious sedation and nitrous oxide inhalation have been proven less popular practices in ASD patients (36).

## The role of the family and Internet information sources

Emphasis should be placed also on training parents in management policies focused on changing the nature or self-perception of the stress-inducing factors. The dental team should aim to navigate parents to accredited professional and social care support networks. Hence, access to rigid scientific evidence will render the parents active participants in therapy decision making and reinforce their confidence in handling the child’s attitude. The Internet has nowadays become the second most popular source of health care consultation for families following their physicians (38). However, due to unregulated and diverse quality of the Internet information and the lack of knowledge of lay persons, there is a high likelihood that users will come across Web sources of doubtful credibility and accuracy (39). Di Pietro and colleagues analyzed the data disseminated by high-traffic autism advocacy sites and concluded that the preponderance of citations about treatment safety and efficacy were unsubstantiated (38). Dental professionals should post on the practice Web site controlled, user-friendly, and reliable data regarding dental treatment of patients with special care needs together with evidence-based education materials.

## Clinical recommendations and future research goals.

**Figure 2:**
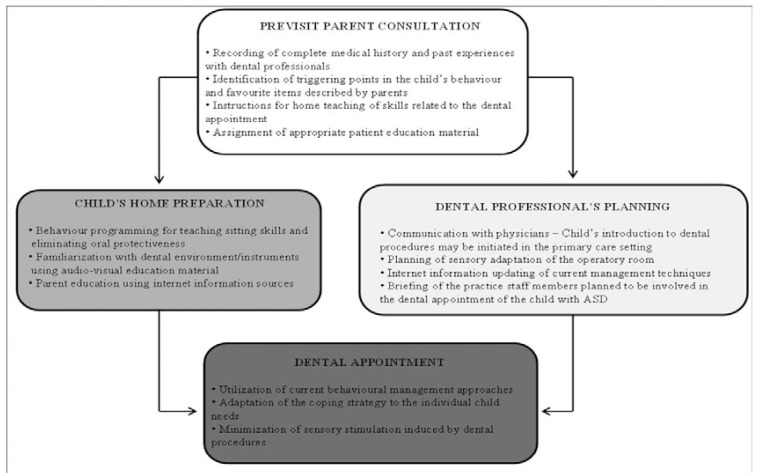
Flow diagram of the procedures suggested to be carried out before and during the dental appointment with the autistic child.

Dental care should be viewed as integral part of comprehensive health care program coordinated by the medical home (40). Based on the higher frequency of the regular medical screening of autistic children compared to scheduled dental visits (19), it can be presumed that an interdisciplinary approach with the child’s physician might help to overcome the anxiety of the dental appointment. Lai and co-authors (19) suggested an oral examination to be planned during primary care attendance to initiate the introduction of the child to dentistry. Successful management of children with ASD requires preparation of the parents and child prior the dental visit, systematic desensitization of the operatory environment, case-by-case adaptation of conventional behavioural methods. The overview of the steps needed to be considered in the dental management of an autistic child is illustrated in figure 2. Under these conditions, dental attendance, initially perceived as a frightening event, can be effectively turned into a controllable experience.

Further insight of the exact association between the primary care provider’s and dentist’ perception of the child’s behaviour in the dental chair should be gained by future interdisciplinary research. Evidence-based coping regimes for patients with ASD also need to be developed to improve compliance with oral care procedures. Taking this for granted, the dental practitioner will be provided with the opportunity to deliver health services in a personalized and appropriate manner.

## Conclusions

The dental management of a child with ASD requires in-depth understanding of the autistic behavioural profile. Based on well-established behavioural guidance techniques, the therapeutic approach should be individualized for each patient. The role of continuous education of dental professionals and parents is essential in overcoming the difficulties encountered by the autistic child in the dental chair.
